# Negative mood induction effects on problem-solving task in women with eating disorders: a multi-method examination

**DOI:** 10.1186/s40337-022-00591-0

**Published:** 2022-05-21

**Authors:** Elan N. French, Kalina Eneva, Jean M. Arlt, Angelina Yiu, Eunice Y. Chen

**Affiliations:** Temple Eating Disorders Program, Temple University, 1701 North 13th Street, Philadelphia, PA 19122 USA

**Keywords:** Eating disorders, Problem-solving, Respiratory sinus arrhythmia, Interpersonal skills, Anorexia nervosa, Bulimia nervosa, Binge eating disorder

## Abstract

**Background:**

The effects of negative affect on problem-solving and its psychophysiological correlates are poorly understood in eating disorder populations.

**Methods:**

This study examined respiratory sinus arrhythmia (RSA) and skin conductance responses of women with Binge Eating Disorder (BED: *n* = 56), Anorexia Nervosa (AN: *n* = 12), Bulimia Nervosa (BN: *n* = 32), and 24 healthy controls (HCs) at baseline, and then during: a negative mood induction task, an adapted Means Ends Problem-Solving (MEPS) task, and recovery. The MEPS task included four interpersonal scenarios: (1) binge-eating as a solution to stress, (2) job loss, (3) rejection by friends, and (4) by a significant other.

**Results:**

We found that individuals with eating disorders reported less positive mood than HCs and individuals with BN and BED reported more negative mood and greater urges to binge than HCs. After a negative mood induction, women with BED provided significantly less effective problem-solving strategies compared to HCs and women with BN for the binge-eating MEPS scenario. Relative to baseline and the negative mood induction, all participants exhibited significantly higher skin conductance measures throughout the MEPS scenarios and recovery. BED showed significantly lower respiratory sinus arrhythmia (RSA) levels than individuals with BN and HCs throughout the protocol.

**Conclusions:**

The multimethod findings suggest individuals with BED are likely to have disorder-specific problem-solving difficulties after a negative mood induction.

**Supplementary Information:**

The online version contains supplementary material available at 10.1186/s40337-022-00591-0.

## Plain English summary

*There is some evidence that individuals with eating disorders experience more negative mood and have challenges with social problem-solving. However, the ability to solve social problems while in a negative mood has not yet been examined while also looking at physiological changes. We measured physiological responses of women with Binge Eating Disorder (BED; n = 56), Anorexia Nervosa (AN; n = 12), Bulimia Nervosa (BN; n = 32), and healthy controls (HCs; n = 24) before and after a task designed to induce a negative mood followed by a problem-solving task. The problem-solving task asked individuals to come up with solutions to social problems related to 1) binge-eating urges, 2) job loss, 3) rejection by friends, and 4) rejection of a significant other. We found that individuals with eating disorders reported less positive mood than HCs and individuals with BN and BED reported more negative mood and greater urges to binge than HCs. Individuals with BED generated fewer effective solutions on a binge-eating problem-solving task and had consistent respiratory sinus arrhythmia differences, which may indicate emotion regulation difficulties.
*

**Supplementary Information:**

The online version contains supplementary material available at 10.1186/s40337-022-00591-0.

## Introduction

Multiple theories on the etiology and maintenance of eating disorders (EDs) have pointed to negative affect as a trigger for engagement in disordered eating (e.g., 1–3) In addition, people with EDs also show greater problem-solving difficulties [4, 5] 

Interpersonal problem-solving draws on both executive functioning abilities and emotion regulation, which both can rely on “top-down” brain pathways. As a result, competition between emotion regulation and executive function may be stronger during periods of intense emotion. Individuals with EDs show cognitive and neurobiological differences compared to healthy controls that contribute to challenges in certain emotional and executive functioning domains, such as impulsivity and cognitive flexibility, areas that have shown to interfere with problem-solving [6–11]. For instance, there is evidence of frontostriatal circuit differences in EDs which may alter emotion self-regulatory skill [12, 13]. In individuals with EDs, there may also be alterations in insula activity, a brain area that integrates emotional and cognitive information with other sensory information and interoceptive cues [6, 7]. For these reasons, individuals with EDs may struggle with tasks that place demands on both emotional and executive functioning systems, and especially in context of disorder-specific problems surrounding eating (e.g., 5). Problems that involve competition of emotional and executive processing systems may be a particular area of weakness for individuals with EDs, even compared to other clinical populations, with one study finding participants with EDs, compared to participants with other psychiatric conditions, had greater difficulties accomplishing goals while upset [14].

Respiratory sinus arrhythmia (RSA) is a proximal measure of vagal tone, which represents the contribution of the parasympathetic nervous system to cardiac regulation, captured through heart rate variability over the respiration cycle [15, 16]. In mentally or emotionally neutral states, the prefrontal cortex inhibits control of the amygdala, and this is associated with increases in RSA [16–18]. However, negative mood states place additional demands on this inhibitory system, and this is associated with reduced RSA. Lower RSA is associated with poor emotion regulation across many clinical populations [19]. After mood induction procedures, individuals with Anorexia Nervosa (AN) and Binge Eating Disorder (BED) showed reduced RSA levels compared to healthy participants [20, 21].

Interpersonal skills tend to be a particular area of weakness for individuals with EDs and thought to play a key role in both the development and maintenance of EDs [22, 23]. Individuals are more reactive to interpersonal stress-related paradigms than control groups [24]. We conducted a systematic review of studies assessing interpersonal problem-solving abilities in ED samples (see Additional file 1: Fig. S1a, b and Table S1c for details). While all 12 included studies [20, 21, 25–34] identified a pattern of ED groups showing difficulties in interpersonal problem solving, there were no studies examining interpersonal problem-solving abilities after a mood induction procedure. Only one study assessed interpersonal problem-solving while concurrently assessing psychophysiological responses. In this study, adolescents with AN, relative to adolescents without AN, had significantly faster heart rate during high-conflict discussions with their mothers compared to baseline [34]. As the systematic review reveals, there is limited published evidence of the effect of problem-solving tasks on individuals with different EDs where psychophysiological response is assessed–with none of the studies assessing RSA concurrently.

The Means Ends Problem Solving (MEPS) task uses realistic, complex problem scenarios to measure the ability to effectively generate solutions to these problems [35]. Individuals with BED, AN, and subclinical disordered eating samples tend to generate less effective solutions on this task [28, 30, 36]. However, the MEPS has not been administered while psychophysiological measures such as RSA have been assessed concurrently.

In the current study, the MEPS task and psychophysiological measures are utilized to examine problem-solving after a mood induction procedure in women with AN, Bulimia Nervosa (BN), BED, and healthy weight women without eating disorders (HCs). Individuals’ autobiographical memories are elicited for the mood induction [37]. Skin conduction level measured physiological arousal [38] during the mood induction and MEPS task. In addition, the MEPS task does not include disorder-specific scenarios. As previous research has shown that individuals with EDs have poorer performance in these domains [5, 20], we included a binge-eating scenario. This was chosen above a restrictive eating or compensatory behavior because urges to binge-eat occur across all ED groups [39].

We hypothesized all ED groups, compared to HC women, (1) would have greater self-reported negative affect, less positive affect, and higher urges to binge-eat in response to a negative mood induction and throughout the protocol. We also hypothesized that (2) women with EDs would generate fewer and less effective solutions compared to HCs, especially on the binge-eating scenario, and that (3) during the negative mood induction and throughout the MEPS task, individuals with EDs would show lower RSA compared to HCs. Comparisons between ED groups were also made, although given the limitations of the literature, we had no specific hypotheses about differences between the AN, BED, and BN groups and these hypotheses should be regarded as exploratory. Findings may have important treatment implications for developing interventions to increase social problem-solving skills, especially in situation that might elicit intense emotional affect.

## Materials and methods

### Participants

Participants were self-referred from the community using flyers, referred from local eating disorder clinics and student health and counseling services, and recruited in a University-based outpatient eating disorder program as part of several clinical trials. The study received approval by the Institutional Review Board of the university.

Inclusions for ED sample included: (1) being medically stable for outpatient treatment as assessed by an external medical exam; (2) being female; (3) aged 18–60 years, and (4) meeting DSM-IV-TR [40] diagnosis of BN or Eating Disorders Otherwise Not Specified criteria for subthreshold BN, and Eating Disorders Otherwise Not Specified criteria for subthreshold and full BED (with individuals meeting subthreshold BED in DSM-IV-TR (40) now meeting full DSM 5 criteria for BED; [41] We also included participants meeting the International Classification of Diseases 10th edition Typical or Atypical AN diagnosis [42] with no current drug or alcohol dependence or symptoms of psychosis. Exclusions for the ED sample included (1) past bariatric surgery or seeking bariatric surgery, (2) in a weight loss intervention currently, or (3) on medications primarily indicated for appetite or weight (e.g., Sibutramine). Other psychotropics, including antidepressants, were acceptable if the dose was stable for at least 3 months prior to screening. HCs were eligible to participate if they did not meet diagnosis for current Axis I disorders [40]. Exclusions for both ED and HC participants included the use of anti-seizure, beta-blockers, motion-sickness medications (e.g., Scopolamine), and asthma medication. These were excluded as they affect cardiac signals.

Prior to beginning the protocol, participants were screened by a Masters-level clinician using the Eating Disorders Examination-16 [43] and the Structured Clinical Interview for Diagnostic and Statistical Manual of Mental Disorders–IV-Text Revision (SCID-IV-TR; 40). The second session consisted of the experimental procedure.

The final included sample consisted of 56 women who met criteria for BED, 12 for AN, and 32 for BN, as well as 24 HC participants without a history of psychiatric disorders (see Table 1 for demographic and clinical characteristics). Overall, group differences in clinical characteristics were aligned with diagnosis status. As expected, the BED group had the highest BMI, followed by HCs and the BN group, and, lastly, the AN group had the lowest BMI (all group differences were significant except the BN vs. HCs). ED groups had greater eating disorder psychopathology (as measured by the EDE subscales) and higher lifetime prevalence of mood and anxiety disorders compared to HCs. Additionally, the AN group had more anxiety disorders than the BED group, and the BN and AN groups scored higher on the EDE dietary restraint scale than the BED groups. Furthermore, the BN and BED groups had a higher rate of medication use and medical problems (such as high blood pressure, asthma, high cholesterol, etc.) compared to HCs, and the BN group also had more medical problems than the BED group. Consistent with clinical presentation, the BED and BN groups engaged in more bingeing than the AN and HC groups. Finally, those with BN had the highest frequency of vomiting episodes than all other groups.

**Table 1:** Sociodemographic and clinical characteristics of the sample

	Anorexia nervosa	Binge eating disorder	Bulimia nervosa	Healthy controls	F	*p*-value
	*n* = 12	*n* = 56	*n* = 32	*n* = 24		
	M (SD)	M (SD)	M (SD)	M (SD)		
Age in years	34.33 (11.60)	41.13 (9.74)	30.19 (9.92)	29.25 (10.90)	11.62	< 0.000
BMI (kg/m^2^)^†^	18.66 (3.94)	36.73 (10.22)	26.13 (6.03)	26.92 (4.63)	25.34	< 0.000
EDE-EC^†^	3.65 (1.77)	2.84 (1.40)	2.74 (1.20)	0.04 (0.08)	35.71	< 0.000
EDE-DR^†^	3.95 (1.41)	2.14 (1.28)	3.14 (1.32)	0.52 (0.92)	28.73	< 0.000
EDE-WC^†^	3.15 (1.77)	3.58 (1.09)	3.45 (1.17)	0.52 (0.69)	45.04	< 0.000
EDE-SC^†^	3.94 (1.34)	3.84 (1.29)	3.72 (1.09)	0.60 (0.81)	49.38	< 0.000
Objective binge episodes^‡^	1.42 (2.67)	4.49 (4.09)	6.55 (6.43)	0.00 (0.00)	12.09	< 0.000
Vomit episodes^‡^	2.26 (4.05)	0.13 (0.94)	7.34 (8.75)	0.00 (0.00)	18.47	< 0.000

	N (%)	N (%)	N (%)	N (%)	φ	*p*-value
Caucasian	10 (91.67)	34 (60.71)	22 (68.75)	14 (58.33)	0.376	0.130Δ
Black	0 (0.00)	8 (14.29)	4 (12.50)	5 (20.83)		
Asian	0 (0.00)	2 (3.57)	0 (0.00)	2 (8.33)		
Mixed	2 (16.67)	9 (16.07)	1 (3.13)	0 (0.00)		
Hispanic	0 (0.00)	3 (5.36)	5 (15.63)	3 (12.50)		
Marital status: single^§^	8 (88.89)	25 (44.64)	19 (59.38)	14 (60.87)	0.244	0.067
Employment status	11 (91.67)	51 (91.07)	29 (90.63)	23 (95.83)	0.071	0.891
Income status^§,‖^	10 (88.33)	17 (30.36)	17 (53.13)	16 (69.57)	0.378	0.001
Mood disorders	7 (58.33)	36 (64.29)	24 (75.00)	0 (0.00)	0.541	< 0.000
Anxiety disorders	9 (75.00)	21 (37.50)	12 (37.50)	0 (0.00)	0.420	< 0.000
Medical comorbidities^§^	7 (70.00)	27 (51.92)	21 (75.00)	4 (21.05)	0.363	0.002
Medication use	6 (50.00)	29 (51.79)	15 (46.88)	2 (8.33)	0.336	0.003

^†^*BMI* body mass index, *EDE* eating disorder examination, *EC* eating concerns, *DR* dietary restraint, WC weight concerns, *SC* shape concerns

^‡^Weekly average within the previous three months

^§^Missing participants' responses for: Marital status (3 AN and 1 HC); Income status: (1 HC); and Medical comorbidity (2 AN, 4 BED, 4 BN, and 5 HC)

^¶^Employment status = Employment / Full-time Student status

^‖^Income status = low-income status (i.e., household income is < $25 000)

ΔValues for racial group differences

### Procedures

#### Negative mood induction

Negative mood was induced through autobiographical memories and sad music, as per the protocol of Hernandez and colleagues [44]. During the negative mood induction, each individual listened on earphones to a compilation of classical music that has been shown to induce sad moods (i.e., unpleasant affect and low arousal; [44, 45] while being instructed to recall their preselected autobiographical memories for 8-min total.

### Measures

#### State measures

**Fig. 1:**
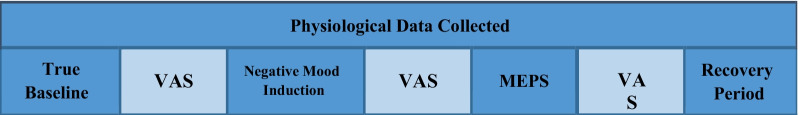
Timeline of Procedure. This starts from baseline to the Means-End Problem Solving (MEPS) to the recovery period with visual analogue scales (VAS) of emotion taken in between time points

Participants reported their current states using the Visual Analogue Scale (VAS; [46]) three times: (1) before and (2) after the negative mood induction, and (3) after the MEPS. See Fig. 1. The VAS ranged from 0 to 100 and assessed the following states: frustration, anxiety, happiness, tension, fear, sadness, and “urge to binge” with higher scores indicating greater emotional intensity.

#### Behavioral measures of problem-solving

The MEPS Task [35] was used to assess problem-solving, where participants were provided with a scenario that describes a problem and a final stated outcome. Participants were instructed to generate the steps taken to achieve the given outcome. In the current study, four sets of MEPS items were adapted from the original task [35]. The scenarios consisted of (1) a situation where binge-eating is seen as a solution to address stress, (2) job loss, and (3) and (4) two situations involving social rejection (one by friends and one by a significant other). The ‘Binge-eating’ scenario was a new scenario developed for the ED population, and presented the story of an individual who considered binge-eating after experiencing a stressful work week and an interpersonal conflict with a relative, and ended with her deciding not to engage in a binge.

The MEPS task gave participants 60 s to describe the most effective strategy for solving the problem and an additional 60 s to describe alternative strategies. For each of the four MEPS scenarios, two dependent variables were derived: the overall effectiveness of the participant’s response*,* which was blindly rated on a 7-point scale (1 being not at all effective to 7 being extremely effective), and the number of relevant means (active problem-solving strategies) the participant produced.

The scoring method was based on the protocol by Williams and colleagues [47], where we only counted active problem-solving related to a specific behavior that were visible to other. For example, in response to the Binge Eating scenario, one participant described:

Other coping methods would just be things that would distract her from those feelings or allow her to cope with them or just in a sense relieve stress. She could [1] draw or [2] paint or [3] sculpt. She could also try [4] journaling, or [5] begin writing a creative story. [6] Exercising would be another good way to get that kind of endorphin boost that would alleviate stress. She could also try just [7] hanging out with her friends or perhaps getting a [8] massage or do some [9] aromatherapy.

This yielded 9 active strategies that were not general—such as distraction from feelings. Participants’ responses were taped and later scored by two independents raters (2 undergraduate students trained by KE), both blind to group status. The 2 raters rated these independently, the 2 sets of ratings reviewed and resolved by KE, in consultation with EYC. Reliability checks between rates yielded coefficients of 0.84 (excellent) for number of relevant means and 0.68 (substantial) for ratings of effectiveness.

#### Psychophysiological measures

We collected electrocardiogram (ECG) data utilizing a modified Lead II configuration. We derived RSA by using the high frequency band of spectral analysis, defined as greater than 0.15 Hz. We measured skin conductance responses (SCRs) and skin conductance levels (SCLs) as measures of sympathetic activity [48] using two electrodes placed on the palm of the non-dominant hand. Tonic SCL refers to level SC collected over each period, whereas SCRs refer to the number of responses per period.

#### Statistical treatment

Statistical analyses were run using SPSS Version 28.01.0. To statistically assess whether the data met assumptions for an ANOVA model, we assessed for normality and for outliers and assessed for homogeneity of variance by assessing for sphericity of the data. Outliers were removed in the event they represented errors in data (e.g., inaccurate psychophysiological data recordings). Sphericity was examined using Mauchly’s test, and Greenhouse–Geisser correction was applied for epsilon values below 0.75 [49]. We estimated power given our results for the purpose of future studies [50] to interpret our results. Partial eta squared (η^2^) was used as the effect size calculation, with 0.01 indicating a small effect; 0.06 indicating a medium effect, and 0.14 indicating a large effect [51].

Six mixed model ANOVAS were conducted using the repeated measures of Condition (baseline, during the mood induction, and during the MEPS) and the between variable of Group (BN, BED, AN, and HC) to assess (1) self-reported negative emotions, (2) happiness, (3) urges to binge-eat, (4) RSA, (5) SCR, and (6) SCL. For each of the four MEPS scenarios, a univariate ANOVA was used to examine group differences in the number of relevant means and effectiveness of solutions for each of the four MEPS scenarios. A Tukey correction was applied for between-subject (Group) contrasts and Šidák for within-in subject (Condition) contrasts.

## Results

### Main analyses

Four extreme outliers were identified for the RSA data, due to inaccurate psychophysiological readings and therefore were excluded from the analysis. Power was above 80% for all significant main effects. If future studies replicate and extend our study design, the sample size used in our current study would be sufficient. Greenhouse–Geisser epsilon values were above 0.75 for all measures, except RSA (epsilon = 0.713), SCR (epsilon = 0.684), and SCL (epsilon = 0.508); therefore, the correction was used for the psychophysiological analyses.

*Self-reported emotions*. Overall, there was a main effect of Condition on emotions, such that all participants reported more negative emotions, *F*_(2, 106)_ = 28.74, *p* < 0.001, η^2^ = 0.21, and fewer positive emotions, *F*_(2, 106)_ = 56.20, *p* < 0.001, η^2^ = 0.35, after completion of the negative mood induction, in comparison to baseline and after the MEPS task. There was a main effect of Group on negative (*F*_(3, 106)_ = 4.27, *p* = 0.007, η^2^ = 0.11) and positive emotions (*F*_(3, 106)_ = 5.50, *p* = 0.002, η^2^ = 0.13). Individuals with EDs reported fewer positive emotions than HC participants. Participants with BN (*p* = 0.005), and BED (*p* = 0.042) reported significantly more negative emotions than HCs. For urges to binge-eating, there was a main effect of Group, *F*_(3, 106)_ = 9.88, *p* < 0.001, η^2^ = 0.22, such that individuals with BED and BN demonstrated significantly greater urges to binge-eat compared to the HC subgroup (*p*’s < 0.001), although there was no effect of Condition. There was no Condition x Group interaction for negative emotions, positive emotions, or urges to binge-eat. See Table 2 for group means and standard deviations.

**Table 2:** Self-reported negative and positive emotions and urges to binge-eat throughout protocol

	NE before induction	NE after induction	After MEPS
	*M (SD)*	*M (SD)*	*M (SD)*
AN	32.49 (26.67)	42.76 (23.27)	44.49 (23.94)
BED	26.95 (20.18)	48.11 (27.90)	32.97 (23.39)
BN	26.73 (21.69)	62.20 (18.90)	35.38 (22.56)
HCs	11.87 (16.43)	38.44 (30.62)	20.31 (21.85)

	PE before induction	PE after induction	After MEPS
	*M (SD)*	*M (SD)*	*M (SD)*
AN	31.02 (22.05)	3.44 (4.35)	20.94 (22.38)
BED	35.91 (23.34)	10.47 (18.50)	27.65 (19.80)
BN	31.60 (24.65)	7.12 (14.69)	23.89 (23.31)
HCs	53.72 (21.58)	20.65 (22.92)	43.52 (28.78)

	UTB before induction	UTB before induction	UTB before induction
	*M (SD)*	*M (SD)*	*M (SD)*
AN	29.78 (36.68)	16.65 (33.35)	23.78 (36.21)
BED	33.65 (31.15)	40.57 (35.50)	35.76 (31.26)
BN	26.98 (27.04)	31.88 (35.34)	34.70 (36.46)
HCs	0.65 (1.81)	0.51 (1.05)	0.43 (0.95)

Missing 14 subjects (3 AN, 2 BED, 4 BN, & 5 HC) for emotion variables due to equipment or software failures

*AN* anorexia nervosa, *BED* binge eating disorder, *BN* bulimia nervosa, *HEs* healthy controls, *MEPS* means end problem solving, *NE* negative emotions, PE positive emotions, *UTB* urge to binge

*MEPS*. A univariate ANOVA suggests that there were significant group differences between effectiveness of the solutions generated for the ‘Binge-Eating’ scenario, *F*_(3,92)_ = 5.95, *p* = 0.001, η2 = 0.16. Women with BED generated significantly fewer effective solutions than HCs and women with BN (*p*’s < 0.03), although there were no differences in the number of relevant solutions generated. There were no differences between diagnostic groups on the remaining three MEPS scenarios (*p’*s > 0.05). See Table 3 for group means and standard deviations.

**Table 3:** Number of relevant and effective solutions in the Means End Problem Solving Scenarios per group

	Amount	Effectiveness
	*M (SD)*	*M (SD)*
Binge eating scenario		
AN	7.75(3.37)	3.75 (1.91)
BED	5.74 (2.45)	3.42 (1.50)
BN	6.40 (2.75)	4.57 (1.48)
HCs	6.80 (2.44)	4.95 (1.28)
Job performance scenario		
AN	5.25 (2.55)	4.50(1.69)
BED	5.26 (2.42)	3.58 (1.54)
BN	4.60 (1.67)	4.03 (1.35)
HCs	4.95 (1.85)	4.15 (0.93)
Friends scenario		
AN	3.88 (1.25)	3.38 (1.77)
BED	4.03 (1.42)	3.18 (1.04)
BN	4.37 (2.22)	3.47 (1.55)
HCs	4.75 (1.59)	4.00 (1.12)
Significant other scenario		
AN	3.88 (1.13)	2.88 (1.13)
BED	4.68 (2.00)	3.61 (1.57)
BN	4.07 (2.07)	3.27 (1.55)
HCs	5.05 (1.99)	4.15 (1.04)

*AN* anorexia nervosa, *BED* binge eating disorder, *BN* bulimia nervosa, *HCs* healthy controls, Number of Relevant Means = Amount

**Fig. 2:**
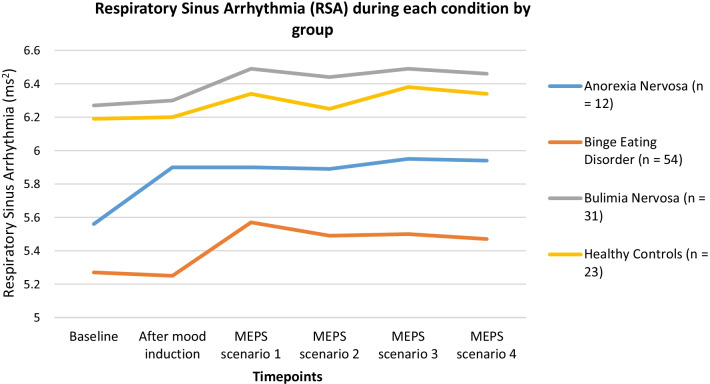
Respiratory Sinus Arrhythmia (RSA) during each condition by group. Means of Respiratory Sinus Arrhythmia Values at: (1) Baseline, (2) Negative Mood Induction, (3) Means-End Problem Solving (MEPS) Scenario 1: Binge eating, (4) MEPS Scenario 2: Job Performance, (5) MEPS Scenario 3: Friends, (6) MEPS Scenario 4: Significant other, and (7) Recovery

*Psychophysiological measures*. There was a significant effect of Group for RSA values (*F*_(3, 116)_ = 5.40, *p* = 0.002, η^2^ = 0.12), such that individuals with BED exhibited significantly lower RSA levels than individuals with BN (*p* = 0.003) and HCs (*p* = 0.023) throughout the protocol. There was a significant effect for Condition, (*F*_(4.28, 116)_ = 4.95, *p* < 0.001, η^2^ = 0.41), such that RSA values were lower during baseline and during the negative mood induction procedure compared to the MEPS scenarios involving urges to binge and rejection by friends. Baseline RSA was also lower than the after the MEPS scenario involving rejection by a significant other. There was no significant Condition X Group interaction. See Fig. 2 and Table 4 for RSA findings.

**Table 4:** Respiratory Sinus Arrhythmia during condition by group

	Anorexia Nervosa		Binge eating disorder		Bulimia nervosa		Healthy controls	
	*n* = *12*		*n = 54*		*n = 31*		*n = 23*	
	*M*	*SD*	*M*	*SD*	*M*	*SD*	*M*	*SD*
Baseline	5.56	1.12	5.27	1.40	6.27	1.22	6.19	1.44
After mood induction	5.90	1.31	5.25	1.36	6.30	1.19	6.20	1.33
MEPS scenario 1	5.90	1.14	5.57	1.22	6.49	1.03	6.34	1.24
MEPS scenario 2	5.89	1.16	5.49	1.28	6.44	1.05	6.25	1.25
MEPS scenario 3	5.95	1.19	5.50	1.36	6.49	0.99	6.38	1.26
MEPS scenario 4	5.94	1.25	5.47	1.28	6.46	1.03	6.34	1.19
Recovery Period	5.99	1.14	5.42	1.35	6.27	1.15	6.28	1.31

There was a significant effect of Condition for SCRs (*F*_(4.11, 87)_ = 39.70, *p* < 0.001, η^2^ = 0.31) and tonic SCL values (*F*_(3.05, 90)_ = 46.09, *p* < 0.001, η^2^ = 0.34). Relative to baseline and the negative mood induction, all participants exhibited significantly higher SCR and SCL levels throughout the MEPS scenarios and during the recovery period (*p*’s < 0.001). Additionally, the binge-eating MEPS scenarios had higher SCR and SCL than all other conditions (*p*’s < 0.03). For SCR and tonic SCL values, there were no significant effects of Group or Condition X Group interaction. SCR and SCL are reported in Additional file 1: Tables S2 and S3 respectively.

## Discussion

This study used a multimethod design to test the effects of a negative mood induction on interpersonal problem-solving in individuals with EDs compared with HCs. Self-reported negative emotions, concordantly with SCR and SCL, increased following a negative mood induction across a sample of women with EDs and HCs, further validating this mood induction procedure in ED samples [52, 53] Additionally, on the binge-eating scenario from the MEPS, the BED group generated significantly fewer effective solutions and had significantly lower RSA than HCs and the BN group. Across groups, RSA values were lower during baseline and during the negative mood induction procedure compared to the MEPS scenarios involving urges to binge and rejection by friends. Baseline RSA was also lower than the after the MEPS scenario involving rejection by a significant other.

### Problem-solving challenges

Following a negative mood induction, while completing the MEPS disorder-specific problem-solving scenario, women with BED generated fewer effective solutions relative to HCs. Consistent with these findings, a systematic review by Kittel, Brauhardt, and Hilbert [54] suggests that women with BED have greater cognitive processing biases compared to normal weight and overweight HCs, in the context of ED disorder-related stimuli (e.g. food). Our study provides further evidence that less effective solution generation after a negative mood induction may be particularly pronounced for ED-specific stimuli in BED. Unlike other eating disorders, meeting criteria for BED in DSM-5 [41] does not require meeting the symptom criterion that an individual’s self-evaluation is based on weight and shape. Overconsumption of food is found in other disorders. Nonetheless, this finding adds to the conceptualization of BED as a psychiatric disorder centered around problems in situations with food and eating.

Contrary to previous studies, we did not observe fewer effective solutions in non-ED-specific MEPs problem-solving scenarios in individuals with BED. While past research demonstrates that individuals with BED can struggle in certain interpersonal domains [23, 55], especially in the presence of negative affect [56], the only previous study using a MEPS task with individuals with BED showed that this group produced less effective solutions on interpersonal scenarios compared to overweight HCs [30]. As the BED group in our study had a significantly higher BMI in our sample, and obesity has shown to negatively influence certain domains of executive functioning, it is possible that obesity compounded the difficulties in problem-solving we observed [57]. More research is needed to confirm if challenges in problem-solving are disorder-specific or more general across interpersonal situations in BED.

Similarly, we did not observe poorer performance in the other MEPS problem-solving scenarios for individuals with BN or AN. This was inconsistent with previous studies assessing interpersonal problem-solving in clinical EDs or individuals with ED symptomology compared to HCs [20, 21, 25, 29, 36, 58], including those using the MEPS.

### Mood induction and psychophysiological results

The BED group exhibited lower RSA compared to the BN and HC groups. Lower RSA is associated with a wide range of psychological problems, including emotional rigidity and poor social functioning [59]. Research suggests that individuals with BED have poorer emotion regulation skills than HC groups [60, 61]. Past psychophysiological experiments using individuals with BED also reported a decreased parasympathetic response to stress [62, 63] although one other study did not show consistent psychophysiological differences [64]. However, our findings fit with a model of reduced RSA being associated with negative mood and stress on the parasympathetic system [18].

### Strengths and limitations

Our study did not include separate control groups of overweight and normal-weight individuals without EDs in order to dissociate the effects of weight. High BMI or other coexisting-medical conditions (such as diabetes and hypertension) may cause blunted cardiovascular reactivity [65, 66], which may have lowered RSA levels in the BED group. However, other studies controlling for weight have shown that women with EDs have greater challenges in emotion regulation [63, 67] and problem-solving [54, 68] domains. As the sample size for the AN group was small, it is more difficult to detect differences between participants in the AN group versus the other groups, and the study design would have been improved by additional participants in this group. Because we did not induce other moods prior to the MEPS, we cannot draw definitive conclusions about whether only negative mood leads to poorer problem-solving.

Study strengths included the use of a disorder-specific problem-solving scenario in a heterogeneous, clinical ED sample compared to a control group and a multi-modal data collection approach. Moreover, the diversity within our sample, particularly within the BED group, is a strength as women of color with EDs tend to be underrepresented in ED research.

## Conclusion & future directions

Our results show significant differences in emotional regulation processes during interpersonal problem-solving in individuals with BED using self-report, behavioral, and psychophysiological data. Individuals with BED may have a cognitive vulnerability to negative affect that may contribute to poorer interpersonal problem-solving, particularly in disorder-specific situations (i.e. binge-eating). Further multi-method studies that capture subjective and physiological experience, as well as behavioral response may help clarify the role of psychophysiology and emotion regulation in the observed interpersonal difficulties common to individuals with EDs.

## Supplementary Information

**Additional file1: Fig. S1a**. Systematic reviewsearch criteria of studies assessinginterpersonal problem-solving in eating disorder samples. **Fig. S1b**. PRISMA flow diagram of studies assessing interpersonal problem-solvingin eating disorder samples. **Table S1c**.Summary of IncludedStudies in Systematic Review. **Table S2**.Skin Conductance Responses at: (1) Baseline, (2) Negative Mood Induction, (3)Means End Problem Solving (MEPS) Scenario 1: Binge-eating, (4) MEPS Scenario 2:Job Performance, (5) MEPS Scenario 3: Friends, (6) MEPS Scenario 4: Significantother, and (7) Recovery. **Table S3**.Mean and Standard Deviations of TonicSkin Conductance Level at: (1) Baseline, (2) Negative Mood Induction, (3) MeansEnd Problem Solving (MEPS) Scenario 1: Binge-eating, (4) MEPS Scenario 2: JobPerformance, (5) MEPS Scenario 3: Friends, (6) MEPS Scenario 4: Significantother, and (7) Recovery.

## Data Availability

The datasets used and/or analyzed during the current study are available from the corresponding author on reasonable request. See code by ENF: https://osf.io/rd8su/?view_only=fac54ba53b094666919a25e32b1d63df.

## Abbreviations

AN: Anorexia nervosa
BN: Bulimia nervosa
BED: Binge eating disorder
EDs: Eating disorders
HCs: Healthy controls
MEPS: Means ends problem solving
SC: Skin conductance
SCL: Skin conductance levels
SCR: Skin conductance responses
RSA: Respiratory sinus arrhythmia
