# Supplementary material for: Negative mood induction effects on problem-solving task in women with eating disorders: a multi-method examination
Source: J Eat Disord. 2022 May 21;10:73. doi: 10.1186/s40337-022-00591-0 (PMC9123706; doi:10.1186/s40337-022-00591-0)
Supplement: Supplementary file 1 — Additional file1: Fig. S1a. Systematic reviewsearch criteria of studies assessinginterpersonal problem-solving in eating disorder samples. Fig. S1b. PRISMA flow diagram of studies assessing interpersonal problem-solvingin eating disorder samples. Table S1c.Summary of IncludedStudies in Systematic Review. Table S2.Skin Conductance Responses at: (1) Baseline, (2) Negative Mood Induction, (3)Means End Problem Solving (MEPS) Scenario 1: Binge-eating, (4) MEPS Scenario 2:Job Performance, (5) MEPS Scenario 3: Friends, (6) MEPS Scenario 4: Significantother, and (7) Recovery. Table S3.Mean and Standard Deviations of TonicSkin Conductance Level at: (1) Baseline, (2) Negative Mood Induction, (3) MeansEnd Problem Solving (MEPS) Scenario 1: Binge-eating, (4) MEPS Scenario 2: JobPerformance, (5) MEPS Scenario 3: Friends, (6) MEPS Scenario 4: Significantother, and (7) Recovery. [file 40337_2022_591_MOESM1_ESM.docx]

**Supplementals**

We conducted a brief systematic review of interpersonal problem-solving in eating disorder samples

**Databases**: PubMed and PsycINFO

**Dates**: until 12/01/2019

**Search terms**:

("eating disorder" OR "eating disorders" OR "Anorexia Nervosa" OR "Bulimia Nervosa" OR "Binge-eating Disorder" OR "Binge Eating Disorder")

AND

("Means Ends Problem Solving" OR "MEPS" OR "Anorexia and Bulimia Problem Inventory" OR "ABPI" OR "interpersonal problem solving" OR "social problem solving" OR "social skills" OR "social intelligence" OR "social abilities" OR "interpersonal skills" OR "interpersonal abilities" OR "communication skills" OR "communication abilities")

**Additional material**: Full dissertations were obtained, when possible, when abstract indicated possible relevant study to this review.

**Inclusion criteria**: (1) an empirical study (2) using individuals with eating disorders (3) that measures interpersonal/social problem-solving skills or other real-life measures of communication skills (4) compared to control group.

**Exclusion criteria**: non-empirical studies (e.g., literature reviews, methods papers, or hypothesis papers), case reports, dissertation abstracts, or articles not in English.

*Supplemental Figure 1a.* Systematic review search criteria of studies assessing interpersonal problem-solving in eating disorder samples.


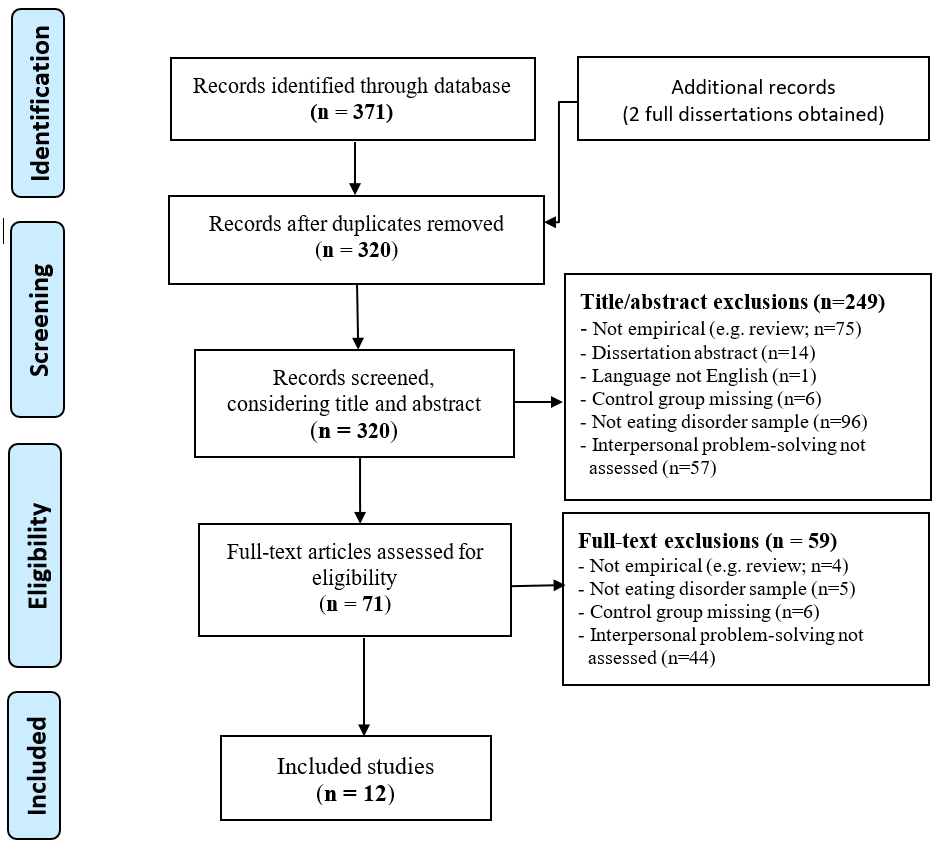


*Supplemental Figure 1b.* PRISMA flow diagram of studies assessing interpersonal problem-solving in eating disorder samples.

*Supplemental Table 1c.* Summary of Included Studies in Systematic Review

| **Citation** | **N** | **Sample group(s)** | **Social task** | **Results Summary** |
| --- | --- | --- | --- | --- |
| Espelage et al., 2000 | 88 | Women with EDs (*n*=44) & HCs (*n*=44) | ABPI | The ED group did worse in the eating and weight, interpersonal relations, academic issues, and family concerns categories of the ABPI subscales. |
| Lattimore et al., 2000 | 34 | Mother-daughter dyads: Adolescents with AN (*n*=20) & psychiatric controls (*n*=14) | PACES | Overall, there was more destructive than constructive communication in the AN group, but not significantly different constructive versus destructive communication in the control group. |
| McAdams & Krawczyk, 2011 | 34 | Women recovered from AN (*n*=17) & HCs (*n*=17) | SPSI-R-SF; fMRI SAT | Recovered AN group did worse than on the SPSI-R-SF; Both groups had similar behavioral performance on social fMRI task, but the recovered AN had reduced activity in the social cognition network. |
| McAdams & Krawczyk, 2013 | 53 | Women with BN (*n*=17), history of AN (*n*=18), & HCs (*n*=18) | SPSI-R-SF; fMRI SAT | Recovered AN and BN groups did worse than on the SPSI-R-SF compared to HCs; All three groups had similar behavioral performance on the SAT. |
| Paterson et al., 2007 | 89 | Female inpatients with AN (*n*=27) & HCs (*n*=62) | SPSI-R | The AN group had a higher negative, lower positive, more avoidant, and more impulsive/careless problem-solving approach than HCs. |
| Paterson et al., 2011 | 105 | Female inpatients with AN (*n*=55) & HC women (*n*=50) | SPSI‐R | The AN group had a higher negative, lower positive, more avoidant, and more impulsive/careless problem-solving approach than HCs. |
| Sternheim et al., 2012 | 70 | Women with AN (*n*=31) & HCs (*n*=39) | SPRT | The AN group produced poorer solutions compared to HCs on the SPRT. |
| Sternheim et al., 2020 | 74 | Women with AN (*n*=30) & HCs (*n*=44) | SPSI-R; MEPS task | The AN group generated less effective solutions on the MEPS and SPSI-R. Once depression, state anxiety, and intolerance of uncertainty were controlled, differences were no longer significant. |
| Svaldi et al., 2011 | 55 | Women with BED (*n*=25) & overweight HC women (*n*=30) | MEPS task | The BED group had less effective and less specific solutions compared to HC in its solutions. |
| Swanson et al., 2010 | 119 | Women with AN (*n*=43) & HC women (*n*=76) | SPSI-R | The AN group had a higher negative, lower positive problem, and more avoidant careless problem-solving approach than HCs. |
| van Buren & Williamson, 1988 | 41 | BN (*n*=12), martially distressed (*n*=14) and non-distressed (*n*=15) couples | Conflict Inventory | Women with BN and martially distressed females used problem-solving skills less often and withdrew from conflict more often than control females. No differences were found for male participants. |
| Van den Broucke et al., 1995 | 63 | AN and BN (*n*=21), martially distressed (*n*=21) and non-distressed (*n*=21) couples | KPI coding system | ED couples lack some of the non-distressed couples' constructive communication, but avoid the destructive communication style of martially distressed couples |

*Abbreviations*. Anorexia and Bulimia Problem Inventory (ABPI); Anorexia Nervosa (AN); Binge Eating Disorder (BED); Bulimia Nervosa (BN); Health Control (HC); Kategoriensystem für Partnerschaftliche Interaktion (KPI); Parent-Adolescent Conflict Evaluation Scheme (PACES); Social Attribution task (SAT); Social Problem-Solving Inventory-Revised (SPSI-R); Social Problem-Solving Inventory-Revised, Short Form (SPSI-R-SF); Social Problem Resolution Task (SPRT)

*Supplemental Table 2*. Skin Conductance Responses at: 1) Baseline, 2) Negative Mood Induction, 3) Means End Problem Solving (MEPS) Scenario 1: Binge-eating, 4) MEPS Scenario 2: Job Performance, 5) MEPS Scenario 3: Friends, 6) MEPS Scenario 4: Significant other, and 7) Recovery

|  | Anorexia Nervosa | | Binge Eating Disorder | | Bulimia Nervosa | | Healthy Controls | |
| --- | --- | --- | --- | --- | --- | --- | --- | --- |
|  | *n=13* | | *n=42** | | *n=21** | | *n=17** | |
|  | *M* | *SD* | *M* | *SD* | *M* | *SD* | *M* | *SD* |
| Baseline | 1.94 | 2.46 | 2.17 | 1.87 | 1.13 | 1.36 | 2.04 | 1.69 |
| After mood induction | 1.94 | 1.71 | 1.99 | 1.85 | 1.52 | 1.20 | 2.01 | 1.45 |
| MEPS scenario 1 | 5.81 | 2.52 | 4.35 | 2.62 | 3.86 | 3.21 | 5.57 | 2.66 |
| MEPS scenario 2 | 4.22 | 2.87 | 3.97 | 2.45 | 3.54 | 2.65 | 4.66 | 3.58 |
| MEPS scenario 3 | 4.15 | 2.69 | 3.77 | 2.73 | 3.25 | 2.73 | 4.53 | 3.11 |
| MEPS scenario 4 | 3.67 | 2.23 | 3.74 | 2.75 | 3.75 | 3.68 | 4.96 | 3.54 |
| Recovery Period | 3.86 | 2.79 | 4.07 | 2.64 | 2.99 | 2.57 | 4.65 | 2.68 |

*Supplemental Table 3*. Mean and Standard Deviations of Tonic Skin Conductance Level at: 1) Baseline, 2) Negative Mood Induction, 3) Means End Problem Solving (MEPS) Scenario 1: Binge-eating, 4) MEPS Scenario 2: Job Performance, 5) MEPS Scenario 3: Friends, 6) MEPS Scenario 4: Significant other, and 7) Recovery

|  | Anorexia Nervosa | | Binge Eating Disorder | | Bulimia Nervosa | | Healthy Controls | |
| --- | --- | --- | --- | --- | --- | --- | --- | --- |
|  | *n=13* | | *n=42** | | *n=23** | | *n=18** | |
|  | *M* | *SD* | *M* | *SD* | *M* | *SD* | *M* | *SD* |
| Baseline | 2.32 | 2.13 | 3.26 | 2.77 | 2.67 | 4.27 | 2.61 | 3.29 |
| After mood induction | 2.02 | 1.63 | 2.09 | 1.76 | 2.12 | 2.98 | 2.57 | 2.31 |
| MEPS scenario 1 | 4.26 | 3.15 | 4.25 | 3.51 | 3.97 | 5.67 | 4.97 | 4.12 |
| MEPS scenario 2 | 5.37 | 3.65 | 5.14 | 3.82 | 5.06 | 6.53 | 6.61 | 4.70 |
| MEPS scenario 3 | 5.20 | 3.93 | 5.05 | 3.71 | 5.26 | 6.94 | 6.10 | 5.07 |
| MEPS scenario 4 | 5.53 | 4.34 | 5.19 | 3.83 | 4.96 | 6.19 | 5.92 | 4.87 |
| Recovery Period | 6.83 | 5.89 | 5.40 | 3.75 | 5.25 | 7.05 | 5.57 | 4.37 |
